# Characterization of the complete mitochondrial genome and phylogenetic analysis of *Epiverta chelonia* (Coleoptera: Coccinellidae)

**DOI:** 10.1080/23802359.2022.2157196

**Published:** 2023-01-01

**Authors:** Yongke Zhang, Lijuan Zhang, Yingchun Lu, Xiahong He, Hongrui Zhang

**Affiliations:** a Plant Protection College, Yunnan Agricultural University, Kunming, China; b Department of Entomology, College of Plant Protection, Henan Agricultural University, Zhengzhou, China; c College of Education and Vocational Education, Yunnan Agricultural University, Kunming, China; d Key Laboratory of Forest Resources Conservation and Utilization in the Southwest Mountains of China Ministry of Education, Southwest Forestry University, Kunming, China

**Keywords:** Epiverta chelonia, complete mitochondrial genome, phylogenetic analysis

## Abstract

*Epiverta chelonia* (Mader 1933; Coleoptera: Coccinellidae) is an important economically and scientifically valuable insect. In this study, the first complete mitochondrial genome of *E. chelonia* was sequenced and characterized using next-generation sequencing techniques. The circular mitogenome of *E. chelonia* consists of 17,347 bp including 13 protein-coding genes (PCGs), 22 transfer RNA (tRNA) genes, two ribosomal RNA (rRNA) genes, and a control region (D-loop). The base composition was AT-biased (75.77%). Bayesian Inference and Maximum likelihood phylogenetic trees strongly supported the monophyly of Coccinellinae. Also, *E. chelonia* was supported as the sister group of *Subcoccinella vigintiquatuorpunctata*, within Epilachninae. Thus, the *E. chelonia* mitochondrial genome will be a fundamental resource for understanding the molecular phylogenetic relationships of the species-rich family Coccinellidae of Coleoptera.

*Epiverta chelonia* (Mader 1933) belongs to the Coccinellid tribe Epivertini (Mader 1933; Dieke 1947), and is a medium to large-sized lady beetle. Its body is regularly oval, length is 4.6–8.1 mm, and width is 4.1–6.4 mm. The surface of the elytra is distinctly brownish-black with regular yellow maculae. Also, the pubescence is moderately dense, long, and yellowish. *E. chelonia* is distributed in southwestern China including the southwestern Sichuan Province, and northwestern Yunnan Province (Pang and Mao 1979; Katoh et al. 2014; Szawaryn et al. 2015; Tomaszewska et al. 2017). It mainly feeds on the flowers, leaves, and stems of the *Anemone* spp. (Ranunculaceae) and *Artemisia* sp. (Asteraceae) (Pang and Mao 1979). It was listed in the “Lists of terrestrial wildlife under state protection, which are beneficial or of important economic or scientific value” by the National Forestry Administration on 1 August 2000 (https://www.forestry.gov.cn/main/3954/content-959027.html). Therefore, the complete mitochondrial genome of *E. chelonia* was analyzed to provide new insights into the phylogeny of Coccinellidae.

In this study, all the specimens were collected from the leaves of *Aconitum carmichaelii* Debeaux in Heqing County, Dali Bai Autonomous Prefecture, Yunnan Province, China (26° 28′32.43″N, 100° 4′3.24″E) in 2021 and were subsequently identified to species using morphology. Ethics approval was not applicable for this study because it did not contain any human participants or vertebrates. The voucher specimens were deposited in the Plant Protection College, Yunnan Agricultural University (https://www.ynau.edu.cn/, Yongke Zhang, zhangyongke99@163.com) under the voucher number GPC-HQ-1. The total DNA was extracted from six *E. chelonia* individuals, which were collected from the same site and host plant, using the CTAB method. Mitogenome sequencing was performed using the Illumina NovaSeq 6000 (Biomarker Biotechnology Co., Ltd, Beijing, China). Raw and clean reads were assembled using the program Getorganelle v. 1.7.1a (Jin et al. 2020). Next, the assembled contigs were aligned with the mitochondrial genome of related species using blastn (BLAST 2.2.30+). Then, the MITOS web and CGView servers were used for gene annotation and mapping of the graphic view of the mitogenomes, respectively (Grant and Stothard 2008; Bernt et al. 2013).

The complete mitochondrial genome of *E. chelonia* was submitted to Genbank (accession number ON209194). It was a closed circular molecule of 17,347 bp in length containing 13 protein-coding genes (PCGs), 22 transfer RNA (tRNA) genes, two ribosomal RNA (rRNA) genes, and a control region (D-loop). The nucleotide composition was AT-biased (A: 39.25%, C: 15.03%, G: 9.19%, and T: 36.52%). The total length of the 22 tRNAs was 1415 bp, with sizes ranging from 55 bp (*trnS1*) to 70 bp (*trnK*); the A + T content ranged from 56.92% (*trnI*) to 90.32% (*trnE*). Also, the two rRNA genes were 1270 bp (*rrnL*) and 813 bp (*rrnS*) in length, and the 13 PCGs had an overall length of 11,052 bp. Additionally, four PCGs (*nad1*, *nad4*, *nad4l*, and *nad5*) were encoded by the minority strand (N-strand) while the other nine were located on the majority strand (J-strand). All the tRNAs in *E. chelonia* were predicted to fold into a typical cloverleaf secondary structure, except for three genes (*trnH*, *trnL1*, and *trnY*) and one gene (*trnS1*), which lacked a TψC loop and dihydrouracil arm (DHU arm) and loop, respectively, and therefore, could not fold into a typical cloverleaf structure. The 16 s rRNA (1270 bp) was located between *trnL1* and *trnV*, and the 12S rRNA (813 bp) resided between *trnV* and D-loop. Their A + T contents were 81.10% and 77.61%, respectively. All the PCGs of *E. chelonia* had the conventional initiation codon for invertebrate mitochondrial PCGs (ATN), except for *cox1* (TTG). Eleven and two of the PCGs stopped with the termination codons TAA (*atp6*, *atp8*, *cob*, *cox1*, *cox2*, *cox3*, *nad2*, *nad4*, *nad4l*, *nad5*, *nad6*) and TAG (*nad1*, *nad3*), respectively.

The phylogenetic analysis was carried out using 13 PCGs of *E. chelonia* and 12 other species (10 Coccinellidae, 1 Salpingidae and 1 Cucujidae). Additionally, two species from Salpingidae and Cucujidae were chosen as outgroups. The 13 PCG sequences were aligned using the MAFFT algorithm in TranslatorX. The partitions and models were estimated using PartitionFinder 2 (Lanfear et al. 2017). Then the maximum likelihood analyses and Bayesian inference analyses were conducted with IQ-TREE (Nguyen et al. 2015) and MrBayes v. 3.2.6 (Ronquist et al. 2012) in PhyloSuite (Zhang et al. 2020), respectively. The two phylogenetic analyses using different methods yielded the same topology, with only some nodal supporting values which were different. The monophyly at the tribe level within the subfamily was strongly supported in the phylogenetic trees. Also, *E. chelonia* as a sister group to *Subcoccinella vigintiquatuorpunctata* was well supported (Figure 1). In terms of the topology, the species could be divided into three clades from the consensus trees: Epilachninae coccinellids clustered together to form the first clade branching from the base of the tree; Scymnini coccinellids and Coccinellidae sp. 1 EF-2015 (unclassified) clustered together to form the second clade; and Coccinellini coccinellids clustered together to form the third clade. These results provide an important basis for further studies on the mitochondrial genome and phylogenetics of Coccinellidae.

**Figure 1.**
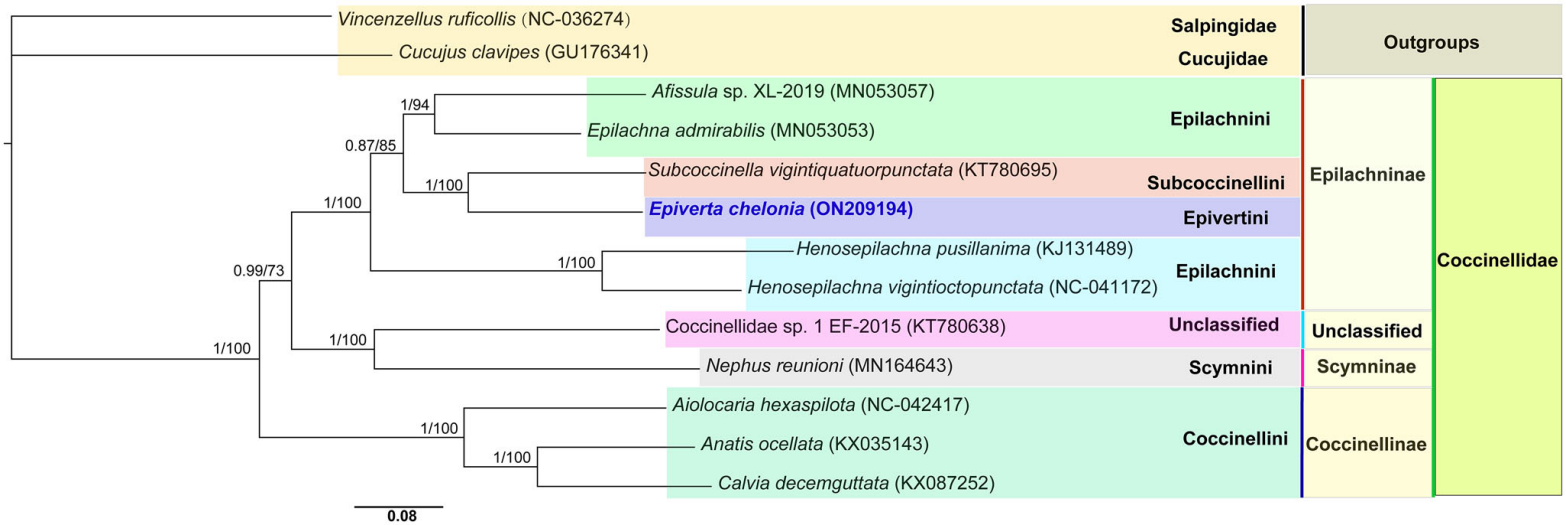
The phylogram was constructed using Bayesian inference (BI) analyses by 2 million generations were run, with 25% of the generations as burn-in. PSRF close to 1.0 and standard deviation of split frequencies below 0.01 were accepted. BI (posterior probabilities) and ML (bootstrap values) support values are reported above nodes, respectively.

## Data Availability

The genome sequence data that support the findings of this study are openly available in GenBank of NCBI at https://www.ncbi.nlm.nih.gov/ under the accession no. ON209194. The associated BioProject, SRA, and Bio-Sample numbers are PRJNA847008, SRR19612121, and SAMN28928437, respectively.
